# Supplementary material for: Gluconate Kinase Is Required for Gluconate Assimilation and Sporulation in Cryptococcus neoformans
Source: Microbiol Spectr. 2022 Apr 12;10(2):e00301-22. doi: 10.1128/spectrum.00301-22 (PMC9045243; doi:10.1128/spectrum.00301-22)
Supplement: SUPPLEMENTAL FILE 1 — Supplemental material. Download spectrum.00301-22-s0001.pdf, PDF file, 0.3 MB [file spectrum.00301-22-s0001.pdf]

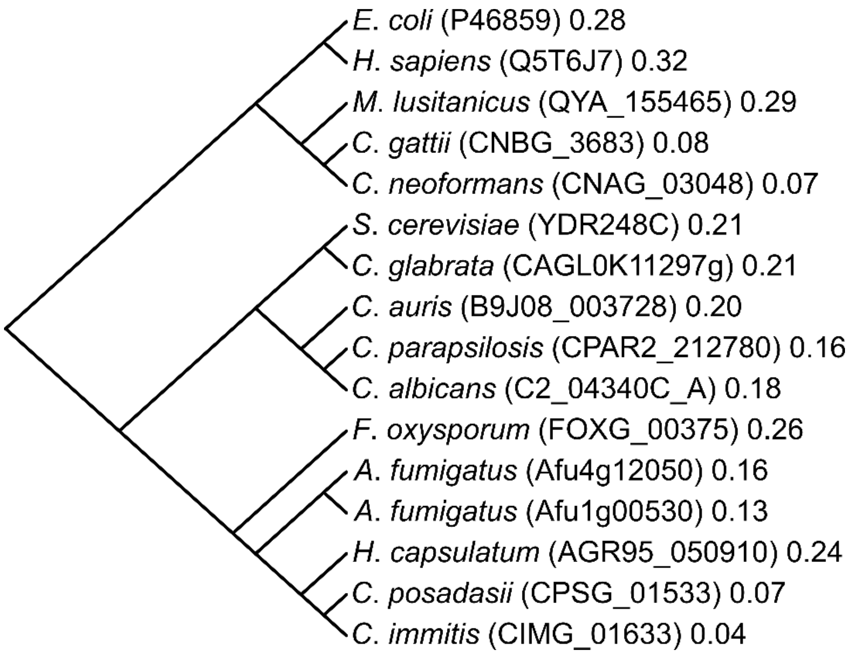

Supplemental Figure 1. Phylogenetic relationship of gluconate kinases among fungal pathogens. Tree that was constructed using Clustal Omega and drawn as a neighbor-joining tree not adjusted for branch lengths using iTOL. Branch lengths to the proximal node are included following the organism and FungiDB protein identifier.

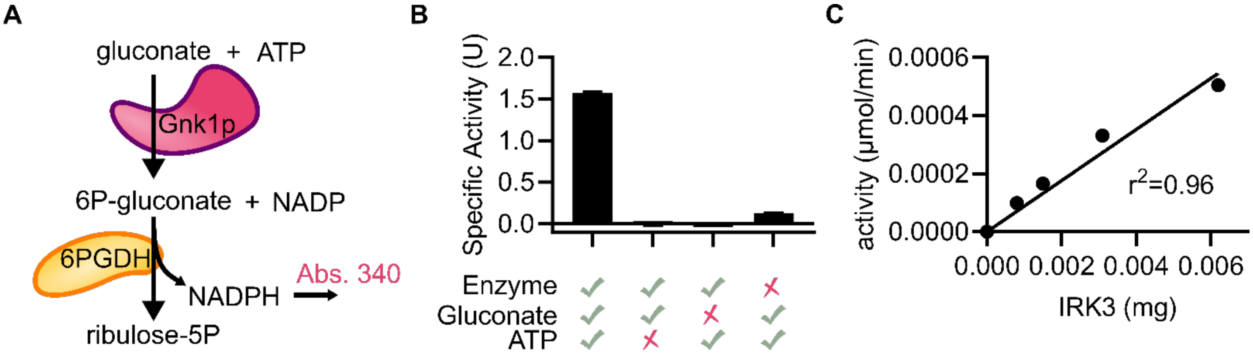

Supplemental Figure 2. Gnk1 activity detection assay validation.

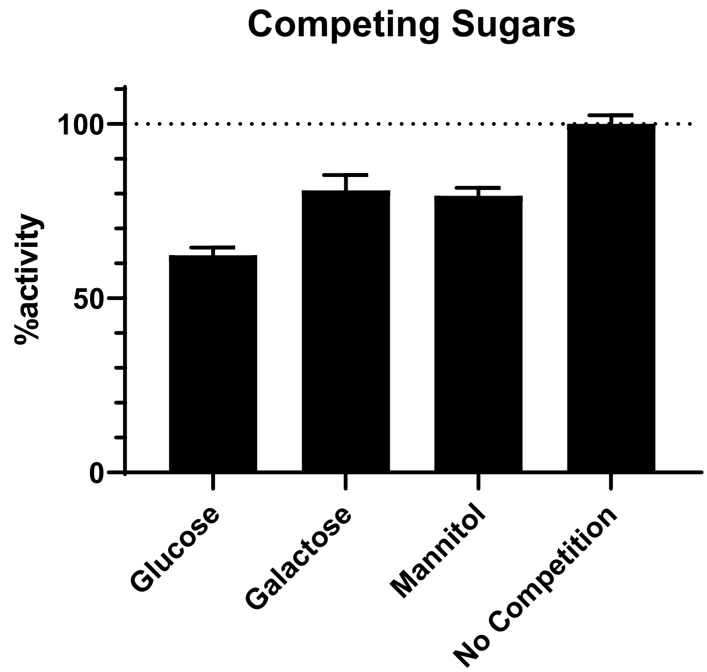

Supplemental Figure 3. Gnk1 prefers gluconate over alternative sugar substrates. Alternative sugar substrates (10 mM) were competed against gluconate (0.5 mM). Activity was normalized to gluconate supplied alone.

No.	Species (locus)	1	2	3	4	5	6	7	8	9	10	11	12	13	14	15	16
1	*A. fumigatus* (Afu1g00530)	-	-	-	-	-	-	-	-	-	-	-	-	-	-	-	-
2	*A. fumigatus* (Afu4g12050)	72	-	-	-	-	-	-	-	-	-	-	-	-	-	-	-
3	*H. capsulatum* (AGR95_050910)	53	51	-	-	-	-	-	-	-	-	-	-	-	-	-	-
4	*C. auris* (B9J08_003728)	43	37	36	-	-	-	-	-	-	-	-	-	-	-	-	-
5	*C. albicans* (C2_04340C_A)	43	41	41	60	-	-	-	-	-	-	-	-	-	-	-	-
6	*C. glabrata* (CAGL0K11297g)	41	39	37	47	45	-	-	-	-	-	-	-	-	-	-	-
7	*C. immitis* (CIMG_01633)	63	59	61	41	44	43	-	-	-	-	-	-	-	-	-	-
8	*C. neoformans* (CNAG_03048)	37	35	30	37	36	35	32	-	-	-	-	-	-	-	-	-
9	*C. gattii* (CNBG_3683)	38	38	34	35	33	39	35	76	-	-	-	-	-	-	-	-
10	*C. parapsilosis* (CPAR2_212780)	42	41	38	59	68	44	40	36	35	-	-	-	-	-	-	-
11	*C. posadasii* (CPSG_01533)	55	54	55	38	40	38	88	30	36	36	-	-	-	-	-	-
12	*F. oxysporum* (FOXG_00375)	57	51	52	47	42	38	56	34	35	43	50	-	-	-	-	-
13	*E. coli* (P46859)	38	37	41	36	34	45	38	35	38	38	34	36	-	-	-	-
14	*H. sapiens* (Q5T6J7)	34	32	32	34	31	35	31	33	31	32	30	37	37	-	-	-
15	*M. lusitanicus* (QYA_155465)	44	37	43	42	41	40	40	40	44	37	38	40	41	39	-	-
16	*S. cerevisiae* (YDR248C)	39	40	39	44	49	59	40	29	30	49	37	40	40	36	39	-

Supplemental Table 1. Alignment matrix of fungal gluconate kinases. Percent similarities of a multiple sequence protein alignment of the indicated gene loci using Clustal Omega multiple sequence aligner. Alignment similarities are shaded such that zero percent similarity corresponds to white and 100 percent alignment corresponds to red.
